# Climate Change and Mortality in Vienna—A Human Biometeorological Analysis Based on Regional Climate Modeling

**DOI:** 10.3390/ijerph7072965

**Published:** 2010-07-21

**Authors:** Stefan Muthers, Andreas Matzarakis, Elisabeth Koch

**Affiliations:** 1 Meteorological Institute, University of Freiburg, Werthmannstraße 10, D-79085 Freiburg, Germany; E-Mail: andreas.matzarakis@meteo.uni-freiburg.de; 2 Central Institute for Meteorology and Geodynamics, A-1190 Vienna, Austria; E-Mail: elisabeth.koch@zamg.ac.at

**Keywords:** mortality, physiologically equivalent temperature, regional modeling, climate change, heat stress, Vienna

## Abstract

The potential development of heat-related mortality in the 21th century for Vienna (Austria) was assessed by the use of two regional climate models based on the IPCC emissions scenarios A1B and B1. Heat stress was described with the human-biometeorological index PET (Physiologically Equivalent Temperature). Based on the relation between heat stress and mortality in 1970–2007, we developed two approaches to estimate the increases with and without long-term adaptation. Until 2011–2040 no significant changes will take place compared to 1970–2000, but in the following decades heat-related mortality could increase up to 129% until the end of the century, if no adaptation takes place. The strongest increase occurred due to extreme heat stress (PET ≥ 41 °C). With long-term adaptation the increase is less pronounced, but still notable. This encourages the requirement for additional adaptation measurements.

## 1. Introduction

The high number of heat-related deaths during the summer of 2003 [1–3], in combination with the probability of an increase of heat wave frequency and duration due to climate change [4] creates the need to assess the future development of heat-related mortality.

The health impact of summer 2003 in Vienna (Austria) was not as severe as in other Western-European countries [5]. In Barcelona (Spain) more than 500 additional people died during the extraordinary conditions of this summer [6] and in Paris (France), where the center of the August heat wave was located [1], more than 300 people died between the 2nd and 12th of August, 2003 [7]. During the summer around 180 deaths were attributable to heat stress in Vienna [5]. In twelve European countries, about 80,000 additional deaths were recorded during the summer [1].

The human body is affected by the thermal environment, which is influenced by many different factors. For heat stress, not only the air temperature, but also the water vapor pressure of the surrounding air, which affects our ability to cool down the body through transpiration, is of importance [8]. Additionally, wind speed can reduce the thermal stress conditions and different radiation fluxes can modify the thermal perception largely [9]. On account for this, an analysis of the relation between heat stress and mortality should include other meteorological parameters besides the air temperature [10].

In addition to the meteorological conditions, physiological parameters of the human body are also relevant. The activity levels affect the internal heat production and the thermoregulation is influenced by different factors e.g., age [8]. Moreover, humans are able to adapt by behavioral measurements, e.g., by their choice of clothing.

Human-biometeorological indexes enable to include all the important factors in the assessment of the thermal conditions. Here, we apply the Physiologically Equivalent Temperature (PET) [11].

The aim of this paper is to analyze the impact of climate change on heat-related mortality. The manuscript is structured as follows: First we present the used data and methods to analyze the relationship between climate and mortality during the past and for the future. Then the results for the past and the possible development in the 21th century, based on regional climate simulation, are presented.

## 2. Data and Methods

We first assessed the relationship between climate and mortality for the years 1970 to 2007. Since the focus is on the impact of heat stress, for each year only the six months from April to October were considered. In the second phase, we used the relationship of the period of examination to estimate the impact of climate change between 2011 and 2100 in Vienna.

### 2.1. Mortality data and methods

For the period 1970–2007, mortality data was obtained from Statistik Austria (National Statistical Service of Austria). The daily values are classified by sex and cause of death according ICD-10 [12]. The total mortality shows a clear seasonal cycle, with higher values in the winter and lower values in the summer [13,14]. Additionally, a distinct long-term trend exists. In the early 1970s around 80 people died each day, whereas in the early 21th century around 50 deaths per day occurred. Hence, a more complex approach was applied to calculate the baseline mortality and to identify days with higher mortality. Koppe and Jendritzky developed a multi-fold smoothing approach for a calculation of the expected mortality [15]. This approach is based on a Gaussian smoother [16], which considers the seasonal variation, but removes long-term trends and day to day variability. The Gaussian smooth extracts the seasonal cycle by a filter of 365 days, so a half year of the mortality data is removed from the beginning and the end of the period of examination. The smoothed curve is modified to reflect the real seasonal cycle of the mortality data. The result describes the expected mortality. Daily percental deviations from the expected mortality were calculated, which are called relative mortality in the following.

### 2.2. Climate station data and methods

The ZAMG (Central Institute for Meteorology and Geodynamics) climate station *Hohe Warte* in Vienna was chosen to be representative for the federal state of Vienna with a population of 1.6 M. To calculate the PET for this station, the meteorological parameters air temperature, relative humidity, wind speed, and cloud cover at 14 CET were used. The global radiation and the mean radiation temperature (T_mrt_) were estimated based on the cloud cover by the RayMan model [17,18] using the approach described in [19].

PET is based on the Munich energy balance model for individuals (MEMI) and enables a thermo-physiological relevant assessment of the thermal conditions [20,21]. Therefore, the meteorological components of the thermal environment are combined with physiological components of the human body, e.g., activity level, age, clothing and others. Consequently, the thermal conditions are described by the air temperature of a standard indoor environment, where the skin and core temperature of the human organism equal to the body parameter in the complex outdoor environment. The usage of a simple value with the unit degree Celsius enables also non professional users to apply and understand the concept of PET [22].

To assess the wind conditions on the human body, the wind speed was reduced to 1.1 m, a height that represents the center of gravity of the human body. The PET for a standard person (male, 35 years, 75 kg, light activity, clothing insulation of 0.9 clo) was estimated using the RayMan model [17,18]. Additionally a flat surface, without modifying surroundings or shading effects, was assumed.

Former studies found a higher impact of heat waves in early summer, compared to later heat waves [23]. This is mainly the effect of short-term adaptation to the thermal conditions, which is a combined effect of physiological acclimatization and behavioral changes. To allow for this effect we used the HeRATE approach [15]. By applying this approach, the upper limit of the thermal comfort grade of PET (23 °C according to [24]) was modified. This modification reflects the thermal conditions of the previous 30 days. Hence, if the previous weeks were very warm, the limit is increased and for cold conditions, the limit is decreased. The heat stress grades above the thermal comfort level (slight, moderate, strong and extreme heat stress with the thresholds 23, 29, 35 and 41 °C) are moved accordingly.

Based on grades of thermo-physiological stress [24], the mean mortality per grade was calculated for the whole period, per decade and per year. Additionally, differences between man and women, as well as different causes of death were analyzed. Based on the decadal and annual assessment, significant changes in the mean mortality per grade were evaluated. Differences in the mean values were assessed by t-tests; trends in the mean mortality were analyzed by fitting linear regression lines to the annual values. The significance of trend line was evaluated by t-tests (t-statistics) for the slope of the regression line and nonparametric Mann-Kendall test [25].

### 2.3. Assessment of the impact of climate change

The impact of climate change on the heat-related mortality was assessed using two regional climate models in the emissions scenarios A1B and B1. We chose the hydrostatic REMO model [26] with a spatial resolution of ∼10 km and the non-hydrostatic CLM [27,28] with a resolution of ∼18 km. For REMO the climate data, at 13–14 CET were selected. For CLM, where only three hour periods were available, the 12–15 CET period was selected. For both models, the mean over nine grid cells [29,30] next to the climate station *Hohe Warte* were extracted. To reduce the air temperature differences between station and model altitude (about 30 m for both models), a vertical correction with 0.65 K/100 m was applied [29,31].

In REMO we used the parameters air temperature, water vapor, wind speed (in 1.1 m), albedo, global radiation and ratio of diffuse radiation. For CLM the same parameters were used besides the radiation term, which was assessed by the cloud cover. Based on these parameters, the PET for each day was calculated using RayMan [17,18]. For a comparison of station and model data, the period 1971–2000 was used; the future development is based on data for 2011–2100.

In the first step, the projected change in the number of days per year for four different grades of thermo-physiological stress was considered. Based on prior results the number of grades compared to [24] is limited to three heat-stress grades (moderate to extreme heat stress) and a large grade of thermal acceptability. The comparison was made relative to the period 1971–2000 and for the three future periods 2011–2040, 2041–2070 and 2071–2100.

To correct the model bias - both models tend to overestimate the high PET—we compared the PET distributions of the models to the distribution of the station values. For each heat stress threshold (29, 35, and 41 °C), the percentile was calculated and compared to the corresponding percentile value for both models. This results in slightly higher threshold in REMO and CLM with the same amount of days above this threshold at the station and in the models.

The assessment of the development of heat related mortality is a difficult and complex topic, since it depends on the levels of adaptations [32]. Two different approaches were applied, to assess the possible range of future sensitivity, *i.e.*, the relation between thermal stress and mortality. The first approach is rather conservative, which applies the mean relation of the period of examination (1970–2007) to the three future periods, no long-term adaptation is assumed. For the three periods, the mean mortality for each heat stress grade is combined with the number of days per year. The product of both results is called cumulated heat-related mortality and describes the cumulated deviations of the mortality from the baseline for a year.

**Figure 1.**
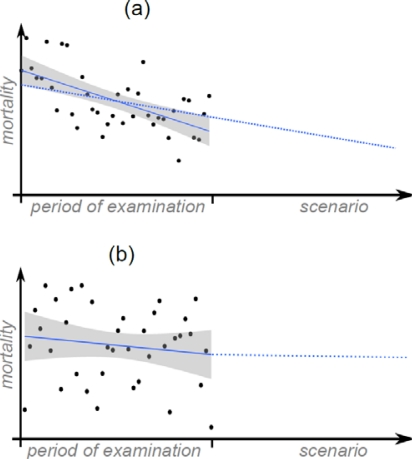
Diagram of the two approaches to assess the future development of sensitivity to thermal stress: Extrapolation of significant (a) and non significant (b) trends (scenario period in relation to the period of examination is not true to scale).

This first approach is probably too pessimistic, since significant decreases in the heat-related mortality in some grades were found in the period of examination. Hence, the mean heat-related mortality per grade for the period 1970–2007 is already too high at the end of this period. To assess the future level of adaptation, significant trends of the period of examination were extrapolated in a statistical conservative manner (Figure 1 (a)), *i.e.*, the lowest declining trend was used. If no significant trend was found in 1970–2007, the sensitivity at the end of the period of examination was used (Figure 1(b)). Since this approach assumes continuous long-term adaptation, it could be too optimistic.

For both approaches, the mean mortality per grade (with or without adaptation) was combined with the number of days per year for each grade and the relative changes in the cumulated heat-related mortality were compared to the values of the period of examination.

## 3. Results and Discussion

### 3.1. The relation of climate and mortality between 1970 and 2007

In the whole period from 1970 to 2007, the mortality on days with PET ≥ 29 °C increases significantly. From April to October, days with PET < 29 °C are characterized by a mean relative mortality of −1.8% (95% confidence interval: −2.1, −1.5). In contrast, on days with moderate heat stress (PET 29–35 °C) a mean relative mortality of 0.9% (CI: 0.4, 1.4) is found and on days with strong (PET 35–41 °C) or extreme heat stress (PET ≥ 41 °C), mortality rises by 5.8% (CI: 5.0, 6.5) and 13.0% (CI: 11.1, 14.7), respectively. The increase is significantly stronger for women compared to men. Additionally, mortality in the combined cause of death group cardiovascular and respiratory diseases (C + R) is higher compared to the all causes mortality, but the differences are not significant (Table 1). The mean relative mortality for the different grades was not constant during the period of examination. In all grades, mortality decreased and in some grades, this decrease was significant.

**Figure 2.**
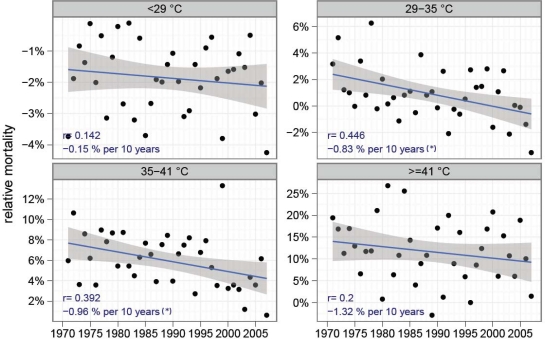
Time series of mean relative mortality for different grades of thermo-physiological stress. Additionally, a linear regression line with CI is included. The parameters of the regression (correlation coefficient and slope) are shown in the lower left corner of each figure. Significant trends at 95% level are marked by (*). Due to the large differences between the thermal stress levels, the y-axes are scaled independently.

Figure 2 presents the mean relative mortality per grade and year, for the years 1971–2006 (1970 and 2007 are omitted, since they contain only a half year due to the Gaussian smoothing in the HeRATE approach).

Days with PET < 29 °C show a slightly decreasing mean mortality (−0.15% per 10 years), but this decrease is not significant (p-value of the slope of the regression line: 0.401; Mann-Kendall: 0.522). On days with moderate heat stress, the decrease is stronger (−0.83%) and the slope of the regression line is significant (CI: −0.68, −0.97, p-value: 0.006 and 0.018). Another significant decrease of −0.96% per 10 years (CI: −0.77, −1.16) was found on days with strong heat stress (p-value: 0.016 and 0.006). The highest-physiological stress grade due to extreme heat stress shows an even higher decrease (−1.32%), but due to the high variability from year to year, the trend is not significant (p-value: 0.234 and 0.187).

The cardiovascular and respiratory diseases group and the overall mortality of women are also characterized by significant trends in the mean heat stress grades. For men, the trend only concerns the 90% significance level on days with moderate heat stress.

### 3.2. Climate change impact

The assessment of the future development is based on two parameters. First the change of heat stress situations, in this case the number of days per grade is of importance. This information is combined with the mean mortality per grade. Two approaches were applied to assess the range of development.

**Figure 3.**
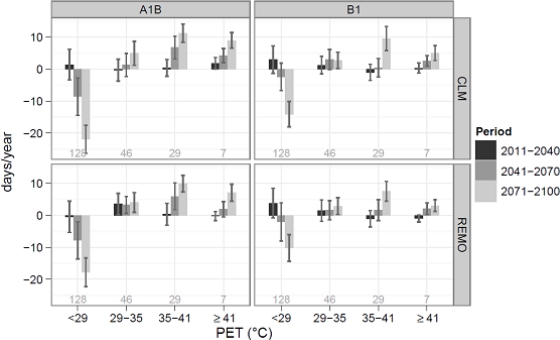
Relative change in the number of days per grade of thermo-physiological stress in two regional climate models and two SRES-scenarios. The changes are relative to 1971–2000, the number of days in the period of examination are shown in gray in the figure.

As shown in Figure 3, no significant change in the number of days per grade compared to 1971–2000 occurs in the first period (2011–2041, except moderate heat stress in REMO-A1B). In the period 2041–2070 heat stress increases significantly in A1B in most of the grades and in B1 in the extreme heat stress grade (+2 to +4 days per year, ∼50%). In the last period (2071–2100) significant increase were found for all heat stress classes, with the highest relative values for days with extreme heat stress. In Vienna, an increase between 3 (42%, REMO-B1) and 9 (129%, CLM-A1B) days of extreme heat stress per year is possible, compared to 7 days between 1971 and 2000. Days with strong heat stress could increase by 28% (+ 8 days) to 39% (+11 days), compared to 29 days in the period of examination.

**Figure 4.**
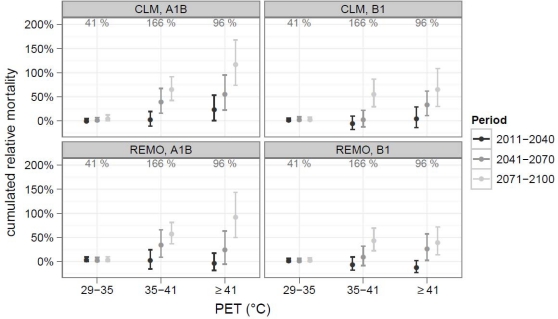
Changes in cumulated heat related mortality without long-term adaptation. The value of the period of examination is shown in gray in the upper margin of each panel.

In the first approach, this change in the number of days per grade is combined with the mean relative mortality between 1970 and 2007, without long-term adaption (Figure 4). No significant increase in heat-related mortality was found for the first period (2011–2040). For 2041–2070 increases were detected in most cases. In the period 2071–2100 heat related mortality could increase by 41% to 121% on days with extreme heat stress and 26% to 39% on days with strong heat stress. Contrary, the changes on days with moderate heat stress are marginal. 100% corresponds to the death cases of a single day without heat stress.

The increases were higher for women as well as for cardiovascular or respiratory diseases, since these groups show a higher sensitivity to thermal stress.

**Figure 5.**
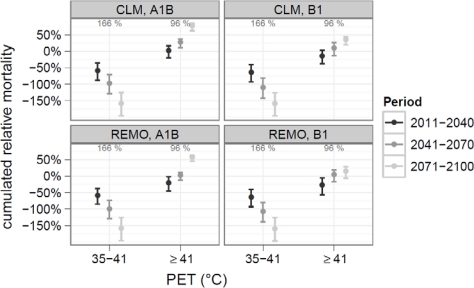
Changes in cumulated heat related mortality including long-term adaptation. The value of the period of examination is shown in gray in the upper margin of each panel. Days with moderate heat-stress were omitted.

A different development is found when long-term adaptation is included (Figure 5). Due to significant trends in the grades of moderate and strong heat stress, mortality on these days decreases continuously. For moderate heat stress, the mean mortality already fell below the baseline at the end of the period of examination. Hence, this grade is omitted in the following text and in Figure 5.

On days with strong heat stress, a statistical conservative trend of −0.77% per 10 years is used. This results in proceeding reduction of heat-related mortality on these days. This reduction is larger, than the increase in the number of days per year. Consequently the cumulated heat-related mortality decreases too. At the end of the 21th century, cumulated mortality on days with strong heat stress is only slightly raised, by 6% in REMO-B1 and 7% in CLM-A1B and it is considerable lower compared to the period of examination (166%).

For mortality due to extreme heat stress, no significant trend was found. Hence, the sensitivity at the end of the period of examination (10.5%) is used. This results in an increase of cumulated heat-related mortality on days with extreme stress. For the period 2041–2070, the increase could be significant at 95% significant level (CLM-A1B). In the period 2071–2100 increases between 15% (REMO-B1) and 77% (CLM-A1B) relative to the 96% of the period of examination were found.

For women, the increase on days with extreme heat stress was higher, with values between 25% (REMO-B1) and 106% (CLM-A1B) for 2071–2100. For the mortality due to cardiovascular or respiratory diseases an increase between 14% and 85% was found.

### 3.3. Discussion

In Vienna, the threshold between heat stress with negative health impacts and thermal acceptability is situated around 29 °C (PET of 14 CET). The differences found between men and women correspond to the results found in other regions: During periods with higher values of thermo-physiological stress, women are statistically significantly (at 95% confidence level) more at risk than men [33–35]. Additional, patient with cardiovascular or respiratory diseases are more sensitive to heat stress [23,36,37].

A significant decrease in mean mortality on moderate and strong heat stress grades was found between 1970 and 2007. This could indicate a successful long-term adaptation process. On days with extreme heat stress, a slight but not significant decrease was found. This could be the effect of a slight adaptation, combined with an increase in the absolute values of thermal stress, since these stress level has no upper limit.

For the assessment of the impact of climate change on human health it is important to include such long-term adaptation trend. Hence, two different approaches, with and without long-term adaptation, were applied. In both approaches the heat-related mortality could increase significantly till the end of the 21th century. Similar trends–increasing heat related mortality even with long-term adaptation, were found for other regions [34,38].

It is well known that older people are very sensitive to thermal stress [2,33,39,40]. Therefore, any assessment of the absolute impact of climate change on heat-related mortality should also consider changes in the demographic structure of a society. In Vienna, the ratio of older people will increase strongly in the following decades [41]. Hence, the absolute number of people that might die due to heat stress, which is masked by the relative mortality in this study, could increase in any case.

To assess the general impact of climate change on the thermal-related mortality, the development of winter mortality should be considered too. Some studies found decreasing cold-related mortality that corresponds with an increase in winter air temperature [42,43]. Hence, global warming could also lead to a reduction of winter mortality [34,44]. Since winter mortality is in general higher than the mean summer mortality, the general effect could also be positive [45]. On the other side are warmer regions at particular risk on cold winters [46], compared to colder regions.

## 4. Conclusions

A significant influence of heat load on mortality was found for the population of Vienna, but this impact has decreased continuously since 1970.

Two different approaches were applied to assess the range of development of heat-related mortality in the future. The upper limit is formed by an approach without any long-term adaptation; the lower limit assumes continuous adaptation.

No significant changes compared to the period of investigation were found for 2011–2040. Hence, time for planning and implementation of additional adaptation measures is available. Measures could e.g., consider the topic of heat stress in urban planning by reducing the radiation component of the thermal environment [47,48].

On a larger scale heat health warning systems could form a promising step, to reduce the health impact of extreme heat waves [49,50]. These measurements should focus on the days with extreme heat stress (PET ≥ 41 °C). These days are characterized by the highest relative mortality and the frequency of these days is likely to increase until the end of the 21th century. Depending on the regional climate model and emissions scenario, increases more than 100% compared to the period of examination were found.

The use of human-biometeorological parameters is recommended to consider all important components of the thermal environment. For a refinement of the results the use of more models or ensembles simulations [51] is planned.

## Figures and Tables

**Table 1.** Mean relative mortality and confidence interval (%) between 1970 and 2007 for different groups and different grades of thermo-physiological stress. (C + R: cardiovascular and respiratory diseases).

**Group**	**PET < 29°C**	**PET 29–35 °C**	**PET 35–41 °C**	**PET ≥ 41 °C**
**All causes**	−1.8 ± 0.3	0.9 ± 0.5	5.8 ± 0.7	13.0 ± 1.7
**All causes–women**	−2.1 ± 0.4	0.8 ± 0.7	6.6 ± 0.9	15.3 ± 2.3
**All causes–men**	−1.3 ± 0.4	1.0 ± 0.7	5.0 ± 1.0	10.4 ± 2.1
**C + R**	−1.8 ± 0.4	1.1 ± 0.7	6.7 ± 1.0	15.7 ± 2.2
**C + R–women**	−2.0 ± 0.7	1.1 ± 1.2	7.3 ± 1.6	17.2 ± 3.6
**C + R–men**	−0.8 ± 0.8	1.0 ± 1.4	5.7 ± 1.9	12.6 ± 3.9
